# Evaluation of the Quality and Reliability of YouTube Videos on Rotator Cuff Tears

**DOI:** 10.7759/cureus.6852

**Published:** 2020-02-03

**Authors:** Tolgahan Kuru, H. Yener Erken

**Affiliations:** 1 Orthopaedics and Traumatology, Canakkale Onsekiz Mart University, Canakkale, TUR

**Keywords:** youtube, rotator cuff tear, discern, jama, video power index

## Abstract

Introduction

The objective of this study was to investigate quality and scientific accuracy of videos related to rotator cuff tear on YouTube.

Methods

Term of “rotator cuff tear” was entered to the searching bar of YouTube and the first 50 YouTube videos about rotator cuff tear with the highest view counts were recorded and evaluated by two orthopedists. Title of the videos that met the inclusion criteria were recorded. The videos were evaluated with DISCERN and JAMA scoring system, and Video Power Index.

Results

The mean DISCERN score was calculated as 35.7±8.9, and the mean JAMA score was found as 2.9±0.4. The mean DISCERN score was statistically significantly higher in the physician group compared to the non-physician group. There was a very strong and statistically significant correlation and an excellent compliance between both observers.

Conclusion

In general, quality of videos published on YouTube about rotator cuff tear was low.

## Introduction

The internet has rapidly become one of the most widespread sources of information, and studies have shown that people use internet to obtain health-related information [1, 2]. Of the people who trust internet based information, 80% are patients with chronic diseases who access health related information online [3]. According to the literature, the internet is currently the first source of medical information for patients with concerns about their disease, and these people use internet to get more information about the disease itself, to search for second options and people who share similar health concerns, to follow up reports on their personal health experiences and even to buy drugs or medical treatment online [4-6]. However, numerous websites contain inaccurate, missing, biased or misleading instructions, making it difficult for the users to make the distinction between reliable and misleading information [7]. Although anyone can access to information via the internet, it may not be possible for everyone to judge quality and accuracy of this information [8].

Today YouTube is the largest media sharing site with 1.0 billion active users monthly, and over 30 million active users daily. The number of YouTube videos viewed daily is ~5 billion. In addition, videos of 300 hours are uploaded to YouTube per minute [9]. YouTube is a valuable source that when used appropriately, could improve learning experience of both public and medical professionals. Distribution of medical information to such a great mass of viewers often involves valuable opportunities, but also dangers because of misleading, even harmful unfiltered videos of poor quality. Therefore, it is important to determine quality and content accuracy of health-related videos on different issues that are published on YouTube. Recently, quality and accuracy of videos about patient education have attracted interest. Most patients believe that health-related information on the internet are equal or even better then the information provided by physicians, and many patients who use internet as a source of information do not report their searching results to their physicians [10].

Health-related videos on YouTube have been evaluated in several studies, and quality of these videos has been generally found as low quality [11-13].

Orthopedics is one of the leading areas for searching on the internet, together with heart failure, mammography, and asthma [14]. In a search made on Google searching engine using “Rotator cuff tear” on 28/03/2019, we obtained 19,600,000 results. Limiting the search with videos, total 177,000 results were found. There are studies evaluating quality and content of YouTube based medical information about various diseases. However, in the literature screening, no study was found about the quality analysis of medical content on rotator cuff tear. Therefore, the objective of this study was to investigate quality and scientific accuracy of videos related to rotator cuff tear on YouTube. From this aspect, our study is first in the literature on this issue.

## Materials and methods

Materials

The term “rotator cuff tear” was entered in the search bar of YouTube (YouTube, www.youtube.com YouTube LLC, San Bruno, CA, USA) at 11/03/2019, and screening was performed by choosing “view count” from the filter options. Among the viewed videos, advertisements, videos with a length of 30 seconds or shorter, those repeated twice or more, and videos with a language other than English were excluded from the study. Title of the videos that met the inclusion criteria were recorded.

The videos included in this study were examined in subgroups as real and animation in terms of the type of image, as physician, health channel, chiropractor, physical therapist, patient, fitness coach and hospital channels in terms of the uploaders, and as general introduction, non-surgical treatment, surgical technique, patient experience, examination techniques and exercise training. In addition, view count, date of upload, comment count, like count, dislike count, and video length of the videos were recorded.

Video Power Index (VPI) values were calculated with the following formula in order to determine popularity of the videos:

[(like count/dislike count + like count) x 100)]

In order to avoid bias from the period of a video on YouTube, the mean daily view count of the videos was calculated according to the following videos:

[total view count determined during viewing of the video by the observers / date of viewing the video by the the observers - upload date of the video to YouTube (days)].

Quality Assessment

Each video with a recorded title was viewed by both observers at the same time, end evaluated with DISCERN (Quality Criteria for Consumer Health Information) and JAMA (Journal of the American Medical Association) scoring systems. DISCERN and JAMA scores were recorded by the observers separately in order to provide objectivity. DISCERN and JAMA scores of the observers were averaged to calculate the mean DISCERN and JAMA scores.

Mean DISCERN score = (DISCERN score of the first observer + DISCERN score of the second observer) / 2

Mean JAMA score = (JAMA score of the first observer + JAMA score of the second observer) / 2

DISCERN Scoring System

The DISCERN tool was used to assess quality of the videos on YouTube. The DISCERN scoring system was developed collectively by the Oxford University and British Library employees, and designed for the use by healthcare consumers. The DISCERN score consists of 15 questions about the content of health information. Users assess the content with a 5-point scale, and total scores differs between 15-75 points. Questions in DISCERN are divided into two sections. The first section (1-8 questions) addresses reliability of the publication, while the second section (9-15 questions) focuses quality of the information about treatment options.

DISCERN scores between 63 and 75 points were classified as ‘excellent’, 51 and 62 as ‘good’, 39 and 50 as average, 28 and 38 as ‘poor’, and <28 as very poor. Higher scores obtained from the scale indicated higher quality of information [15] (Table 1).

**Table 1:** DISCERN Scoring System

DISCERN SCORING SYSTEM						
Section	Questions	No	Partly			Yes
Reliability of the publication	1. Explicit aims	1	2	3	4	5
	2. Aims achieved	1	2	3	4	5
	3. Relevance to patients	1	2	3	4	5
	4. Source of information	1	2	3	4	5
	5. Currency (date) of information	1	2	3	4	5
	6. Bias and balance	1	2	3	4	5
	7. Additional sources of information	1	2	3	4	5
	8. Reference to areas of uncertainty	1	2	3	4	5
Quality of information on treatment choices	9. How treatment works	1	2	3	4	5
	10. Benefits of treatment	1	2	3	4	5
	11. Risks of treatment	1	2	3	4	5
	12. No treatment options	1	2	3	4	5
	13. Quality of life	1	2	3	4	5
	14. Other treatment options	1	2	3	4	5
	15. Shared decision making	1	2	3	4	5

JAMA Scoring System

This system is a quality scale used for evaluation of information obtained from the health-related internet sites. It consists of 4 criteria of “Authorship, Attribution, Disclosure, Currency”. Each item is evaluated with 0 (does not meet the desired criteria) or 1 point (meets the desired criteria). The minimum score that can be obtained from these scale is 0 and maximum score is 4 points. Higher scores obtained from the scale shows increased quality of the information, which is assessed [16] (Table 2).

**Table 2:** JAMA Scoring System

JAMA SCORING SYSTEM		Rating	
Section		No	Yes
Authorship	Authors and contributors, their affiliations, and relevant credentials should be provided	0	1
Attribution	References and sources for all content should be listed clearly, and all relevant copyright information should be noted	0	1
Disclosure	Website “ownership” should be prominently and fully disclosed, as should any sponsorship, advertising, underwriting, commercial funding arrangements or support, or potential conflicts of interest	0	1
Currency	Dates when content was posted and updated should be indicated	0	1

Statistical Analysis

Data of the study was analyzed using SPSS 20.0 statistical package software and expressed as number, percentage, mean ± standard deviation, median, minimum and maximum values. Comparison of the mean DISCERN and JAMA scores in the physician and non-physician groups was made using Mann-Whitney test among the non-parametric tests according to the results of normality test. In order to evaluate correlation between DISCERN and JAMA points, Spearman correlation analysis was used according to the results of normality test. In evaluation of the correlation coefficient, r:0-0.24 was considered as poor, r:0.25-0.49 as moderate, r:0.50-0.74 as strong, and r:0.75-1.0 as very strong. The Cronbach α value was calculated to evaluate the compliance between the observers. Cronbach α < 0.5 was considered as inacceptable, 0.5 ≤ α <0.6 as poor, 0.6 ≤ α<0.7 as acceptable, and 0.7 ≤ α <0.9 as excellent. p<0.05 values were considered statistically significant.

## Results

When general features of the videos were analyzed, we found 42 video contained real images, 14 videos were shared by a physician, and 24 by physical therapist. A majority of the video contents were about exercise training (n=18, 36%), and general information (n=11, 22%) (Table 3) (Figüre 1).

**Table 3:** General features of the examined videos

General features of the videos		
Variables	n	%
Image type		
Real	42	84.0
Animation	8	16.0
Uploaders		
Physician	14	28.0
Health channel	7	14.0
Chiropractor	2	4.0
Physical therapist	12	24.0
Patient	4	8.0
Fitness coach	8	16.0
Hospital channel	3	6.0
Video content		
General information	11	22.0
Non-surgical treatment	3	6.0
Surgical technique	7	14.0
Patient experience	6	12.0
Examination techniques	5	10.0
Exercise training	18	36.0

 

**Figure 1:**
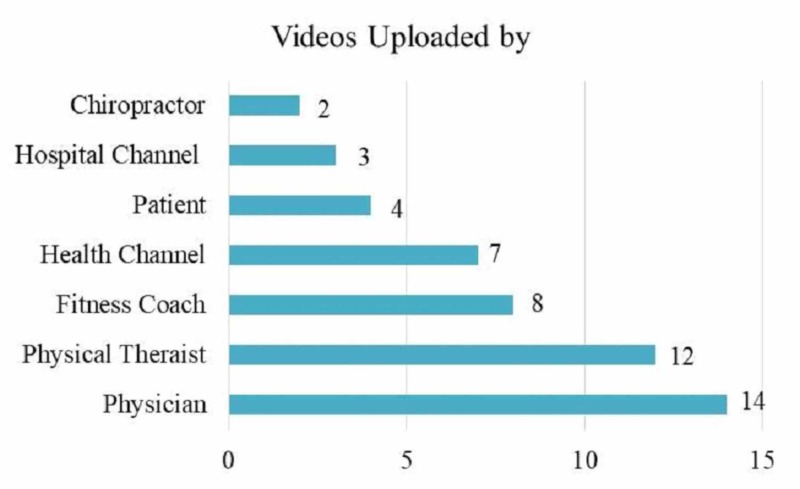
Distribution of the video publishers

Video lengths, view counts, days since uploading, view counts (daily), comment counts, like counts, dislike counts and Video Power Index (VPI) values are given in Table 4. Distribution of the video features according to the uploaders is shown in Table 5.

**Table 4:** Parameters of Video Power Index (VPI)

Variables	Mean±Standard Deviation	Median (Minimum-Maximum)
Video length (minutes)	7.56 ± 6.50	5.77 (0.65-35.17)
View count	401329.0 ± 255850.0	160238.0-1287652.0
Time since video upload (days)	2288.1 ± 1001.7	2538.5 (511.0-4235.0)
View count (daily)	230.5 ± 215.1	162.6 (49.3-1101.9)
Comment count	176.2 ± 243.2	88.5 (0.0-1333.0)
Like count	1811.2 ± 1787.1	978.5 (0.0-7300.0)
Dislike count	106.1 ± 106.6	77.0 (0.0-507.0)
VPI (Video Power Index) (%)	90.6 ± 15.1	93.4 (0.0-98.2)

 

**Table 5:** Distribution of the video features according to the uploaders

	Number of Videos	Video Length (minutes)	Mean		
			Like	Dislike	Comment
Physician	14	6,74	1600	95	86
Physical Terapist	12	5,65	1890	126	248
Fitness Coach	8	7,03	3584	118	315
Health Channel	7	6,84	730	55	28
Patient	4	16,9	1314	122	372
Hospital Channel	3	3,4	285	70	71
Chiropractor	2	11,95	1658	213	95

The DISCERN score of the videos given by the first observer was 35.7± 8.9 and the DISCERN score given by the second observer was 35.8±8.8. The JAMA score of the videos given by the first observer was 2.9±0.4 and the JAMA score given by the second observer was 2.9±0.5. Accordingly, the mean DISCERN score was calculated as 35.7±8.9, and the mean JAMA score was found as 2.9±0.4. 

**Figure 2:**
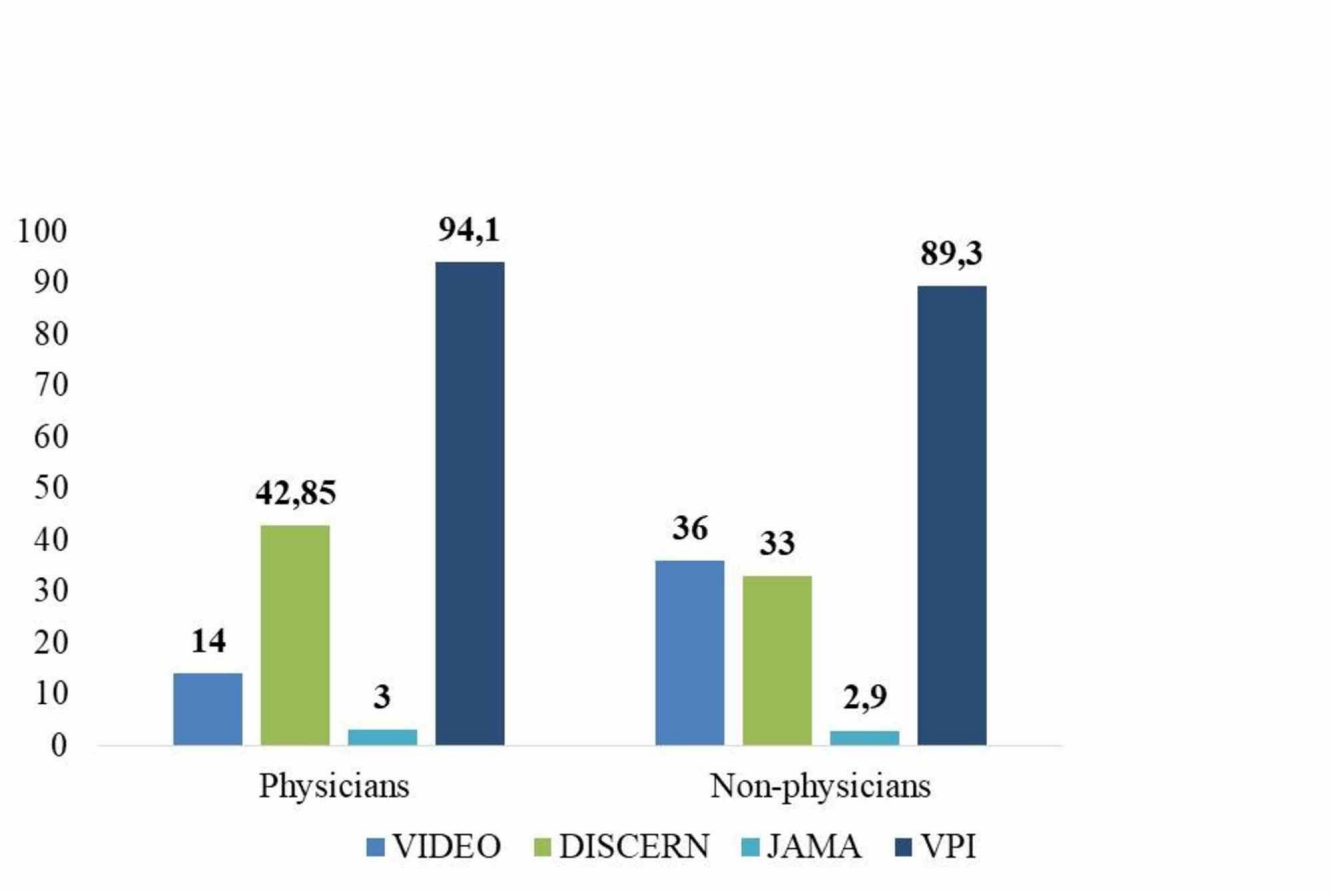
Number of videos, DISCERN, JAMA and VPI values of physician and non-physician groups.

DISCERN, JAMA and Video Power Index (VPI) values of the observers were compared between the physician and non-physician groups. The mean DISCERN score was found as 42.8±8.9 points in the videos shared by a physician, and 33.0±7.3 in the videos uploaded by non-physician person or institutions. Accordingly, mean DISCERN score was statistically significantly higher in the physician group compared to the non-physician group (p=0.001). There was no statistically significant difference between physician and non-physician groups in terms of the mean JAMA scores and VPI values (Figure 2).

When DISCERN scores of the two observers were examined with Spearman correlation analysis, a very strong and statistically significant correlation and an excellent compliance between both observers was found (r:0.997, p<0.001, Cronbach α = 0.998).

Similarly, when JAMA scores of the two observers were examined with Spearman correlation analysis, a very strong and statistically significant correlation and an excellent compliance between both observers was found (r:0.894, p<0.001, Cronbach α = 0.938).

**Figure 3:**
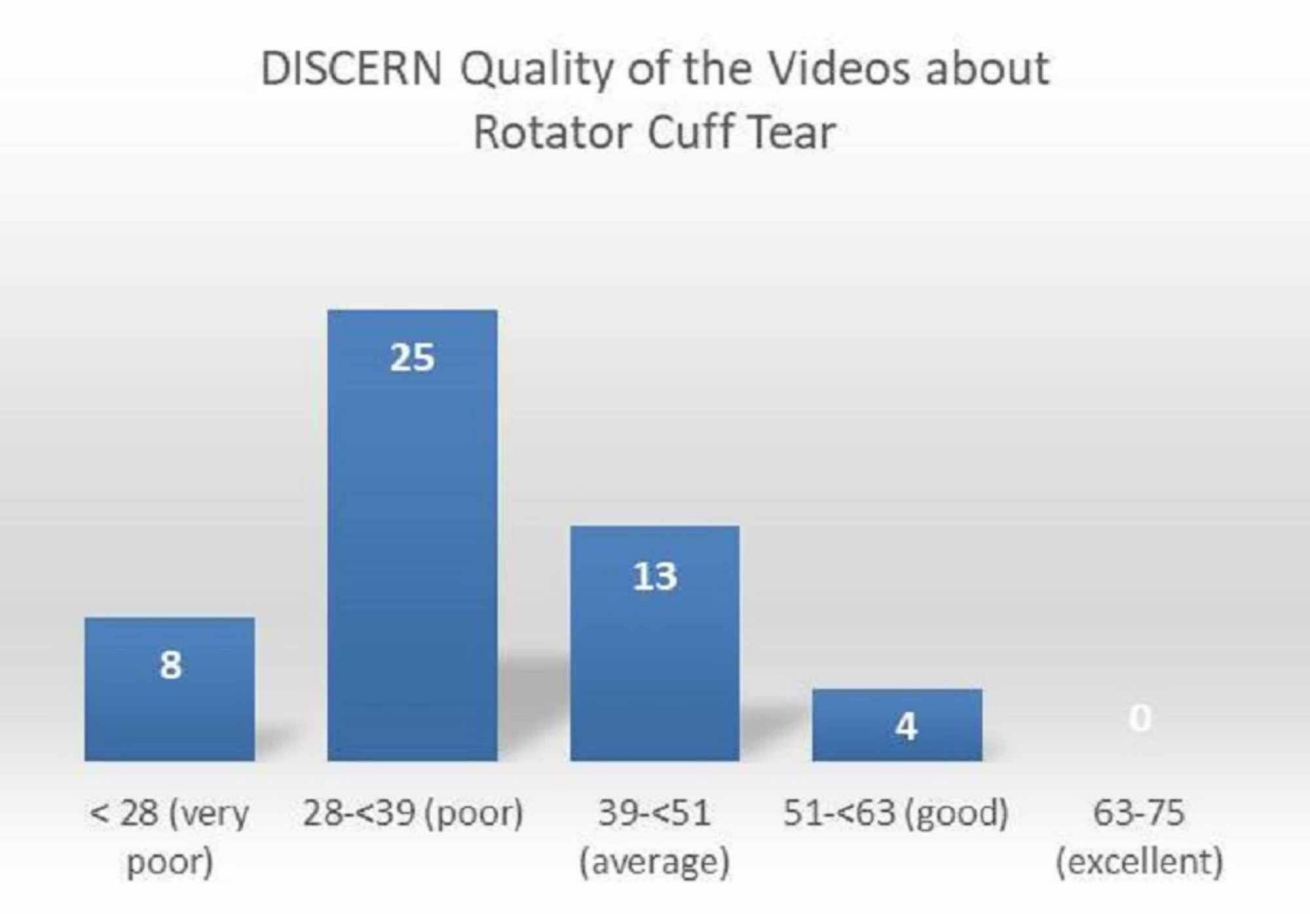
Distribution of quality of the videos according to the DISCERN scoring system

According to the mean DISCERN scores of the two observers, quality of the videos was found as very poor in 16%, poor in 50%, average in 26%, and good in 4% (Figure 3).

## Discussion

Rotator cuff tears are one of the most common pathological conditions of the shoulder, with increasing incidence in patients aged over 50 years, and a progressive pattern in most of the cases [17]. Although arthroscopic rotator cuff repair is one of the most frequently used orthopedic procedures, evidence for the decision are still limited [18]. There are options for treatment of rotator cuff tear according to the classification include surgery, rehabilitation, medical therapy, injections, massage and exercise. Patients who fear from surgery usually refer to health related information-sharing sites on the internet, and especially to YouTube in order to seek alternative treatments. It has been reported that approximately 4.5% of all searches on the web are related to health. About 6.75 million health related searches are performed daily in Google alone [19]. However, majority of health related shares on the internet are made by non-physician persons and/or institutions. Among these shares, patient experience, advertisements, alternative treatment techniques and commercial shares take an important place. Most of these shares contain misleading and even risky information. A useful video contains accurate information on epidemiology, treatments, and procedures of diseases, while a video will be misleading if it contains inaccurate information or promotes treatments that have not been scientifically proven [20].

In our study we aimed to investigate information and content quality, and reliability of the videos about rotator cuff tear on YouTube. This study is the first in the literature, investigating this issue. Consistent with the literature, most of the videos examined were uploaded by non-physician persons. Of the 50 videos, 72% were uploaded by non-physicians with 24% shared by physical therapists, 16% by fitness coaches, 14% by health channels, 8% by patients, 6% by hospital channels, and 4% by chiropractors. Increase in the videos uploaded by non-physicians is not specific to our study, and in a recent systematic review, a considerable part of health related YouTube videos contain anecdotal information and patient experiences [3].

Of the 50 videos evaluated, 8 (16%) were animation and 42 (84%) were real images. The animations were commonly uploaded by health channels. It has been proposed that patients find animation videos more useful. In our study, the rate of animation videos was lower than reported in the literature. We think that this difference between the studies is due to the subject which is being searched. Since studies on this issue are relatively new and limited, studies conducted on the same subject are scarce.

In the present study, mean length of the videos was 7.56 minutes. Previous studies have reported the mean video length between 6.17-10.35 minutes [8, 21]. Our results were consistent with the literature.

Quality and reliability of the first 50 YouTube videos about rotator cuff tear that met the inclusion criteria were generally low. Various scoring systems have been reported in literature in order to evaluate quality and reliability of videos on the internet [20, 22]. In our study, we used DISCERN and JAMA scoring system that are among the most frequently used scoring systems. In our study, the mean DISCERN score was found as 35.7±8.9, mean JAMA score as 2.9±0.4, and Video Power Index rate as 90.6±15.1%. According to the DISCERN scoring, quality of the most video contents was in ‘poor’ category. The mean DISCERN scores (42.8 vs 31.3), JAMA scores (3.0 vs 2.9) and Video Power Index (93.8 vs 89.3) values were higher in the videos uploaded by physicians compared to those uploaded by non-physicians. However, only the difference between DISCERN scores of the videos shared by physicians and those shared by non-physicians reached statistical significance (p<0.001). Similarly, previous studies have also found that quality of the medical information published by physicians was higher than the medical information published by non-physicians [23]. In our study, scores given to the question about reliability and treatment information in the DISCERN scoring system were also higher in the videos shared by physicians compared to the videos shared by non-physicians. However, it has been argued that such videos may not be understandable for patients and thus, may have a low view count [24]. Although in the present study, DISCERN scores were significantly higher in the videos uploaded by physicians compared to those uploaded by non-physicians, quality of these videos was in ‘average’ category. This result suggests that quality and content of such videos uploaded by physicians may not be sufficient, and similar results were reported in the literature [11].

Since previous studies have reported that quality of YouTube videos on various medical and surgical issues was generally low, the findings of our study are not surprising [8, 11-13]. A significant part of the videos were evaluated as very poor/poor by the observers, and compliance between the observers was found as excellent, indicating that most persons who make health searches cannot distinguish low-quality medical information from good-quality medical information. In this point, such studies have a risk for misleading patients with rotator cuff tear. Berland et al. reported that patients who use the internet for medical information may have difficulty to access “exact and accurate information”, and proposed that this missing online health information may negatively affect decisions of patients [25]. In addition, this result revealed that health professionals and health institutions are insufficiently represented in publication of medical information videos.

In our study, there was a negative correlation between video quality and like count. This reflects that high-quality videos are not as popular as low-quality videos. Furthermore, various studies in the literature have reported that useless videos are more popular than useful videos [26-28].

When contents of the videos included in our study were analyzed, the most common content was found as exercise training followed by general information. Rate of the videos containing patient experience was lower than reported in the literature. In our study, only 6 of 50 videos contained patient experience. DISCERN and JAMA scores of these videos were lower than the mean value, as expected. Forty-three of 50 videos contained non-surgical information. This result may be attributed to the patients who search for non-surgical options for the treatment of rotator cuff tear. Finally, we believe that health professionals should be promoted for uploading accurate and reliable contents, that will correctly guide patients, to the media share platforms.

Study Limitations

First, possibility of different results in a search at different times may be considered as a limitation of this study. However, this was a cross-sectional study, and an instant search model was created. Second, viewing only first 50 videos may be evaluated as a limitation. Herein, we included the first results that were often referred, similarly to the literature because, when evaluating from the viewpoint of patients who are not health professionals, patients seem to view the information which they encounter first. In our study, we analyzed only the most viewed videos, and not all videos on this subject on YouTube. Finally, although we performed a comprehensive analysis, we could not evaluated potential association of comments with video length, quality, like and dislike counts. However, use of two different but commonly used methods (DISCERN and JAMA scoring systems) reflects strength of this study.

## Conclusions

In our study, quality of videos published on YouTube about rotator cuff tear was mostly low. In addition, our findings indicate that patients cannot make the distinction between accurate and inaccurate medical information on YouTube, and even give higher rate to low-quality videos. We believe that evaluation of health of the consumers’ behaviour patterns by health institutions, social media platforms and relevant public organization will be important. As a result of such evaluation, strategies could be developed in order to improve quality of medical information provided by YouTube and other internet platforms. This study is first in the literature to evaluate quality of videos about rotator cuff tear. We think that further studies to be performed on orthopedics and other medical areas on this issue would provide contribution to quality, accuracy, and reliability of health related video contents.

## References

[1] AlGhamdi KM, Moussa NA (2012). Internet use by the public to search for health-related information. Int J Med Inform.

[2] Griffiths F, Cave J, Boardman F (2012). Social networks--the future for health care delivery. Soc Sci Med.

[3] Madathil KC, Rivera-Rodriguez AJ, Greenstein JS, Gramopadhye AK (2015). Healthcare information on YouTube: a systematic review. Health Informatics J..

[4] Powell J, Inglis N, Ronnie J, Large S. (2011). The characteristics and motivations of online health information seekers: cross-sectional survey and qualitative interview study. J Med Internet Res.

[5] van Uden-Kraan CF, Drossaert CH, Taal E, Seydel ER, van de Laar MA (2009). Participation in online patient support groups endorses patients empowerment. Patient Educ Couns.

[6] Atkinson NL, Saperstein SL, Pleis J. (2009). Using the internet for health-related activities: findings from a national probability sample. J Med Internet Res.

[7] Young SD (2011). Recommendations for using online social networking technologies to reduce inaccurate online health information. Online J Health Allied Sci.

[8] Gokcen HB, Gumussuyu G (2019). A quality analysis of disc herniation videos on YouTube. World Neurosurg.

[9] https://www.omnicoreagency.com/youtube-statistics/ [vieved: 28/03/2019 (2020). YouTube by the numbers: stats, demographics & fun facts. https://www.omnicoreagency.com/youtube-statistics/.

[10] Diaz JA, Griffith RA, Ng JJ, Reinert SE, Friedmann PD, Moulton AW (2002). Patients’ use of the Internet for medical information. J Gen Intern Med.

[11] Fischer J, Geurts J, Valderrabano V, Hugle T (2013). Educational quality of YouTube videos on knee arthrocentesis. J Clin Rheumatol.

[12] Ho M, Stothers L, Lazare D, Tsang B, Macnab A (2015). Evaluation of educational content of YouTube videos relating to neurogenic bladder and intermittent catheterization. Can Urol Assoc J.

[13] Mukewar S, Mani P, Wu X, Lopez R, Shen B (2013). YouTube and inflammatory bowel disease. J Crohns Colitis.

[14] Drozd B, Couvillon E, Suarez A (2018). Medical YouTube videos and methods of evaluation: literature review. JMIR Med Educ.

[15] Charnock D, Sheppard S, Needham G, Gann R (1999). DISCERN: an instrument for judging the qualityof written consumer health information ontreatment choices. J Epidemiol Community Health.

[16] Silberg WM, Lundberg GD, Musacchio RA (1997). Assessing, controlling, and assuring the quality of medical information on the internet. JAMA.

[17] Omid R, Lee B (2013). Tendon transfers for irreparable rotator cuff tears. J Am Acad Orthop Surg.

[18] Mather RC, Koenig L, Acevedo D (2013). The societal and economic value of rotator cuff repair. J Bone Joint Surg Am.

[19] Eysenbach G and Kohler C (2003). What is the prevalence of health-related searches on the world wide web? qualitative and quantitative analysis of search engine queries on the Internet. AMIA Annu Symp Proc.

[20] Fat MJL, Doja A, Barrowman N, Sell E (2011). YouTube videos as a teaching tool and patient resource for infantile spasms. J Child Neurol.

[21] Ovenden CD, Brooks FM (2018). Anterior cervical discectomy and fusion YouTube videos as a source of patient education. Asian Spine J.

[22] Haymes AT, Harries V (2016). ‘How to stop a nosebleed’: an assessment of the quality of epistaxis treatment advice on YouTube. J Laryngol Otol.

[23] Tartaglione JP, Rosenbaum AJ, Abousayed M, Hushmendy SF, DiPreta JA (2016). Evaluating the quality, accuracy, and readability of online resources pertaining to hallux valgus. Foot Ankle Spec.

[24] Desai T, Shariff A, Dhingra V, Minhas D, Eure M, Kats M (2013). Is content really king? an objective analysis of the public's response to medical videos on YouTube. PLOS one.

[25] Berland GK, Elliott MN (2001). Health information on the Internet: accessibility, quality, and readability in english and spanish. JAMA.

[26] Kumar N, Pandey A, Venkatraman A, Garg N (2014). Are video sharing web sites a useful source of information on hypertension?. J Am Soc Hypertens.

[27] Garg N, Venkatraman A, Pandey A, Kumar N (2015). YouTube as a source of information on dialysis: a content analysis. Nephrology.

[28] Lee JS, Seo HS, Hong TH (2014). YouTube as a source of patient information on gallstone disease. World J Gastroenterol.

